# The Impact of Chromosomal Mosaicisms on Prenatal Diagnosis and Genetic Counseling—A Narrative Review

**DOI:** 10.3390/jpm14070774

**Published:** 2024-07-21

**Authors:** Mariela Sanda Militaru, Ioana-Mădălina Babliuc, Vanesa-Larisa Bloaje-Florică, Valentin-Adrian Danci, Iulia Filip-Deac, Enikő Kutasi, Vasile Simon, Mihai Militaru, Andreea Cătană

**Affiliations:** 1 Department of Molecular Sciences, Faculty of Medicine, University of Medicine and Pharmacy “Iuliu Hatieganu”, 400012 Cluj-Napoca, Romania; mariela.militaru@reginamaria.ro (M.S.M.); andreea.catana@reginamaria.ro (A.C.); 2 Regional Laboratory Cluj-Napoca, Department of Medical Genetics, Regina Maria Health Network, 400363 Cluj-Napoca, Romania; 3 Department for Mother and Child Health, Pediatric 1, Emergency County Hospital, No. 68 Motilor Street, 400394 Cluj-Napoca, Romania; ioana_babliuc@yahoo.com (I.-M.B.); valentindanci1999@yahoo.com (V.-A.D.); simonvasile89@yahoo.com (V.S.); 4 Emergency Clinical Hospital Bagdasar-Arseni, 12 Berceni Road, 4th Sector, 041915 Bucharest, Romania; vanessa.larisa@yahoo.com; 5 County Emergency Clinical Hospital, 50 Dr. Gheorghe Marinescu Street, 540136 Târgu Mureș, Romania; iulia.filip77@yahoo.com; 6 Department of Urology, University of Medicine and Pharmacy “Iuliu Hatieganu”, 11 Tăbăcarilor Street, 400139 Cluj-Napoca, Romania; 7 Pediatric 2 Discipline, University of Medicine and Pharmacy “Iuliu Hatieganu”, Emergency County Hospital, No. 3-5 Clinicilor Street, 400535 Cluj-Napoca, Romania; militarumihai@yahoo.com; 8 Department of Oncogenetics, Institute of Oncology, “Prof. Dr. I. Chiricuță”, 400015 Cluj-Napoca, Romania

**Keywords:** chromosomal mosaicism, prenatal diagnosis, genetic counseling, NIPS/NIPT

## Abstract

Genetic disorders represent a high-impact diagnosis for both patients and their families. Prenatal screening methods and, when recommended, genetic testing allow parents to make informed decisions about the course a pregnancy is going to take. Although offering certainty about the potential evolution and prognosis of the pregnancy, and then the newborn, is usually not possible, genetic counseling can offer valuable insights into genetic disorders. Chromosomal mosaicisms are genetic anomalies that affect only some cell lines in either the fetus or the placenta or both. They can affect autosomal or heterosomal chromosomes, and they can be either numerical or structural. The prognosis seems to be more severe if the genetic alterations are accompanied by malformations visible in ultrasounds. Several genetic techniques can be used to diagnose certain mosaicisms, depending on their nature. A novel approach in prenatal care is non-invasive prenatal screening (NIPS), also known as non-invasive prenatal testing (NIPT), which, although it does not always have diagnostic value, can provide valuable information about potential genetic anomalies, especially numerical, with high sensitivity (Se).

## 1. Introduction

Prenatal diagnosis is a crucial aspect of modern obstetrics, allowing for the early detection of malformations and genetic disorders [1]. Prenatal genetic testing is indicated in cases of ultrasound or biochemical screening abnormalities, a family history of genetic disease, advanced maternal age, and in vitro fertilization (IVF) [2]. 

Chromosomal mosaicism, the presence of distinct cell lines with different chromosomal complements, can arise from errors in meiosis and/or mitosis [3]. Depending on which chromosome is affected, the mosaicism can be autosomal or heterosomal. The aneuploid cells can be present only in the fetus or only in the placenta or it can affect both, depending on the differentiation stage at which the mosaicism occurs [4]. The most common form of chromosomal mosaicism involves monosomy X [5].

The detection and interpretation of chromosomal mosaicism in cytogenetic diagnostics can be challenging due to the presence of cultured artifacts, pseudomosaicisms, and other factors [6]. A range of techniques are used in the diagnosis of chromosomal mosaicism, each with its advantages and limitations. Karyotyping, chromosomal microarray analysis (CMA), and fluorescence in situ hybridization (FISH) are commonly used in prenatal diagnosis [5]. Next-generation sequencing (NGS) platforms, such as MiseqTM Veriseq and Ion Torrent Personal Genome Machine PGMTM ReproSeq, have been shown to accurately detect chromosomal mosaicisms and segmental aneuploidies in embryos preimplantation [7]. 

A range of samples can be analyzed for chromosomal mosaicism in prenatal diagnosis, including amniotic fluid, chorionic villi, and peripheral blood cells [8]. The samples can sometimes be contaminated with maternal tissue fragments, as well as blood or placental cells. When mosaicism is diagnosed using amniotic fluid, the relative proportion of abnormal amniotic cells cannot be precisely correlated with that in fetal tissues [9].

NIPT, or non-invasive prenatal testing, is a method for detecting fetal chromosomal abnormalities by analyzing cell-free fetal DNA, which represents 3–13% of the total circulating DNA in the maternal plasma. The fetal fraction is influenced by biological factors: it increases with gestational age and decreases with a higher maternal BMI and age but is not significantly affected by fetal aneuploidy [10,11,12]. It is a highly sensitive and specific screening tool with a low false positive rate, making it an attractive alternative to traditional serum screenings and invasive tests [13,14]. 

## 2. Autosomal Mosaicism

Prenatal genetic diagnosis and clinical management in cases of chromosomal mosaicism (CM) should be performed with caution because the level of mosaicism and the distribution patterns of abnormal cell lineages can be shifting and unpredictable. For these reasons, different specimens or testing methods should be analyzed and compared in order to formulate a clear overview of the current knowledge on this matter [14].

Zhang et al. [15] performed a single-center retrospective study to evaluate the occurrence and clinical importance of CM in prenatal genetic diagnosis using G-banding karyotyping and chromosomal microarray analysis (CMA). The frequency and clinical features of CM were analyzed in fetuses with and without phenotypic anomalies in ultrasounds by CMA and G-banding karyotyping. The results showed that the most common type of CM was mosaic autosomal trisomy (19.23%, 20/104), and its frequency was greater in fetuses presenting an unusual phenotype (28.85%, 15/52) compared to fetuses with a normal phenotype (9.62%, 5/52). The mosaic fractions were similar between cases with or without phenotypic abnormality based on the general classification or specimen sources. Conflicting mosaic results were obtained in 16 cases (15.38%, 16/104) from distinct specimens or using various testing methods [15].

Even though quantitative fluorescence polymerase chain reaction (QF-PCR) using short tandem repeat (STR) markers is specialized in detecting whole chromosome trisomies of chromosomes 13, 18, 21, X, and Y, partial deletion or chromosomal mosaicism may also be identified. Therefore, QF-PCR can facilitate the identification of deletions or duplications in STR loci, but additional analysis using next-generation sequencing (NGS) or CMA is mandatory to validate the diagnosis [16].

Trisomy 16 (T16) is frequently encountered in the case of first-trimester abortion. Since complete T16 is not compatible with life, the majority of cases are mosaic trisomies. In this context, non-invasive prenatal testing (NIPT) offers both a quick and prompt prenatal screening for chromosome abnormalities and therefore may guide pregnancy management. Peng et al. [17] conducted a retrospective assessment of 14 cases that presented a high risk of T16 by NIPT evaluation (Table 1). Out of all cases, 12 cases were identified as T16 and 2 cases as T16 mosaicism. Using invasive prenatal diagnosis (karyotyping and CMA), five true positive cases and nine false positive cases were confirmed. All nine false positive case pregnancies were continued, and eight out of nine newborns presented low weights (less than 2.5 kg) at birth. There were also two premature deliveries. In conclusion, by combining both cytogenetic techniques and molecular methods, T16 mosaicism can be accurately identified [17].

Multiple genetic tests can be used in detecting fetal trisomy 9 mosaicism. Ma et al. [18] reported two cases of trisomy 9 mosaicism that were initially suggested by NIPT. An elevated level (from 42% to 50%) of mosaicism was obtained after karyotype analysis of the amniocytes in both cases. Uncultured amniocytes were analyzed by CMA, and no CNV was found, besides a large fragment loss of heterozygosity. The ultrasound findings revealed no phenotypic abnormalities except for small size for gestational age. In case 1, CMA and fluorescent in situ hybridization (FISH) performed on uncultured fetal cord blood confirmed trisomy 9 mosaicism. After comprehensive genetic counseling, the parents requested the termination of both pregnancies. Further molecular genetic tests of tissue samples from the aborted fetus and the placenta were performed. The tests revealed the presence of fetoplacental mosaicism. In various tissues, the levels of trisomy 9 mosaicism ranged from 76% to not present [18]. 

Trisomy 8 mosaicism (T8M), also called Warkany syndrome, represents a rare chromosomal disorder. Cell-free fetal DNA (cffDNA) screening was used to initially identify T8M in a 17-week pregnancy. Karyotype analysis, single-nucleotide polymorphism array (SNP-array), FISH, and BACs-on-Beads™ (Bobs™) assay were performed to evaluate the fetal sample and to confirm the diagnosis. After karyotyping, the cultured amniocytes, trisomy 8 was found in 1 of 73 metaphases. SNParray was performed further on cultured amniocytes and neonatal cord blood cells to reveal the existence of T8M. Interphase FISH carried out on native neonatal cord blood cells validated the T8M percentage as 10%. The Bobs™ assay supported the previous findings. The parents were informed about the possibility of fetal defects later on, but they decided to continue the pregnancy. At birth, the baby was normal. A follow-up was carried out at 3 years old. He had language retardation, facial asymmetry, and low-set ears and had experienced periodic fever [19]. 

When it comes to prenatal counseling for autosomal mosaicism, a clear understanding of cell-based chromosome tests’ performance in contrast with CMA and DNA-based copy number variation sequencing (CNV-seq) analyses is necessary to obtain an accurate assessment of autosomal mosaicism levels, especially in detecting low-level mosaicism. In this regard, Ma et al. [4] conducted a retrospective analysis on 5367 pregnancies, and 72 fetuses presented with mosaic chromosomal aneuploidy, including 22 cases with autosomal mosaicism and 5 fetuses with large cryptic genomic rearrangements. Low-level mosaicism was identified in 13 out of 22 cases using CMA, while CNV-seq analyses identified mosaicism levels down to 5% in 19 of the 22 fetuses with autosomal mosaicism (Table 1). Moreover, in all 19 cases confirmed by CNV-seq, the percentages of trisomic cells for autosomal trisomy 21, 18, and 13 were in accordance with the karyotyping results. Nevertheless, the percentage of aneuploidy for cases 16, 17, 18, 19, and 22 was considerably lower in the culture samples in comparison with the uncultured ones. Although trisomy 8 was not identified by karyotyping methods in case 18, CNV-seq, along with CMA, showed 18% and 24% mosaic trisomy 8. Lastly, for cases 11, 20, and 21, the CMA and CNV-seq results were normal in the uncultured amniotic fluid cells, but karyotyping revealed mosaicism for trisomy 9, trisomy 21, and trisomy 20 in cultured amniotic fluid cells. Although CNV-seq proved superior in its effectiveness in identifying further and clinically relevant information concerning autosomal mosaicism compared with CMA, prenatal genetic evaluation of autosomal mosaicism remains a demanding process. According to Ma et al. [4], if karyotype analyses show low-level mosaic aneuploidy, the results should be corroborated with DNA-based tests, preferably CNV-seq, if possible [4].

Liu et al. [20] investigated both the precision and depth evaluation of clinical CNV-seq, also known as low-pass genome sequencing (LP GS), in the identification of chromosomal mosaicism and copy number variants (CNVs). They demonstrated that the uniquely aligned high-quality reads (UAHRs) influenced the detection sensitivity of LP GS for CNVs and mosaic aneuploidies. Consequently, exactly 30 million UAHRs (single-end 35 bp) were suggested to detect mosaic aneuploidies and to identify the majority of mosaic CNVs with values over 1.48 Mb containing mosaicism levels greater than 30%. Therefore, they concluded that CNV size had an impact on the accuracy of LP GS in detecting CNVs and especially in identifying mosaic aneuploidies [20]. 

Concerning the prenatal detection of small supernumerary marker chromosomes (sSMCs), CMA and fluorescence in situ hybridization (FISH) have proven to be key instruments in discovering the origin and the genetic structure of sSMCs. Traditional cytogenetic methods, such as G-banding analysis, provide scarce information regarding the origin of sSMCs. Sun et al. [21] presented the case of an sSMC(15) female fetus who inherited the mosaic sSMC(15) from her mother. G-banding analysis was performed through amniocentesis, and the karyotype of the fetus was 47,XX,+mar/46,XX. Through molecular genetic tests in both the mother and the fetus, the sSMC was identified as inv dup(15) (*D15Z1*++, *SNRPN*-, *PML*-), which proved the maternal inheritance of sSMC(15). The female infant was born with no phenotypic signs of abnormality at 39 weeks of gestational age. Therefore, when it comes to prenatal sSMCs cases, prenatal genetic counseling can be performed effectively using molecular genetic techniques [21].

Prenatal genetic counseling in cases of de novo balanced reciprocal translocation continues to be challenging. Chen et al. [22] reported the incidental identification of a familial 8p23.2 microduplication including *CSMD1* correlated with a karyotype of 46,XY,t(7;8)(q31.2;p23.1)/46,XY diagnosed by amniocentesis in a normally evolving pregnancy. The male baby had no phenotypical modifications at birth. A follow-up checkup was performed at the age of six months, and the infant presented no phenotypic or developmental abnormalities [22].

Although Optical Genome Mapping (OGM) is recognized for its ability to detect chromosomal anomalies, various aberrations are not detected by OGM. In this regard, a study has been conducted aiming to identify the aberrations overlooked by OGM and evaluate the factors involved. The results showed that OGM may miss structural variations (SVs) including CNVs, balanced translocations, and inversions that have breakpoints placed in large repetitive sequences, apart from Robertsonian translocations. According to the authors, GRCh38 is considered the appropriate option as the reference genome when OGM genome assembly is used. In certain circumstances, different genetic techniques used in combination with OGM may improve the detection rate of the method [23]. 

One study assessed eight fetuses with fetal cardiac rhabdomyoma (CR) and their families. Six fetuses out of eight had not previously presented any *TSC1*/*TSC2* variants, and the other two families were suspected of gonadal mosaicism. NGS was performed using tissue from the umbilical cord, CR tissue, and parental blood. All positive results, including two paternal semen samples, were confirmed using droplet digital polymerase chain reaction (ddPCR). The results showed that the fetuses carried low-level somatic mosaic variants, and the CR tissue obtained from one fetus presented a second-hit variant (Table 1). Thus, hybrid capture NGS attained by associating NGS with ddPCR improved the accuracy of prenatal tuberous sclerosis complex (TSC) diagnosis [24].

Although there are only a few cases of mosaic trisomy 12 reported prenatally and postnatally in the literature, prenatal genetic diagnosis of this mosaic aneuploidy can be of great importance [25]. Bonasoni et al. [26] presented a case of mosaic trisomy 12 discovered by amniocentesis, associated with a negative prenatal ultrasound. The family decided to terminate the pregnancy at 22 weeks of gestational age. Postmortem, the fetus displayed non-lethal morphological characteristics not reported previously in the literature. Although the fetal ultrasound may be normal, prenatal genetic counseling should focus on identifying minor abnormalities and the widespread presence of trisomic cell lines in different internal organs, especially in cases of advanced maternal age [26].

## 3. Heterosomal Mosaicism

Sex chromosome abnormalities range from 0.5 to 0.71% of all prenatal diagnoses and can cause fetal gonadal dysgenesis and structural and functional abnormalities of other organs, as well as intellectual disability of varying severity [27,28].

Compared to prenatal diagnosis of the common trisomies (21, 18, and 13), the diagnosis of sex chromosome aneuploidies (SCAs), excluding 45,X0 and its mosaic variants, remains problematic due to the lack of clear clinical and ultrasound abnormalities [29,30].

In a large study comprising 17,428 singleton pregnancies with no structural abnormalities seen in fetal ultrasounds that underwent non-invasive prenatal testing (NIPT), 202 samples were positive for chromosomal anomalies. Out of these, 91 samples showed positive results for SCAs. Most cases were represented by the following chromosomal formulas: 45,X0 (41), followed by 47,XXY (17), mosaic SCAs (12), 47,XXX (11), and lastly 47,XYY (10). As a confirmation procedure, amniocentesis was performed in 78 of the 91 SCA cases, showing there were only 38.46% true positive SCAs. The most frequent true positive rate was seen for mosaic sex chromosome aneuploidies (83.33%), followed by 47,XYY (57.14%), 47,XXY (37.50%), 47,XXX (36.36%), and 45,X0 (28.95%) [31].

Xu et al. [32] conducted a study including 32,931 women with singleton pregnancies who underwent NIPT screening, out of which 140 results were positive for SCAs. Prenatal diagnosis through amniocentesis or cordocentesis was performed in 103 cases. The general PPV (positive predictive value) for NIPT in SCAs was 55.34%, with the individual PPVs ranging from 85% for 47,XXX and 85% for 47,XXY to 68.75% for 47,XYY and only 26.09% for 45,X0. The authors concluded that NIPT should only be used as a screening method, and SCAs observed on NIPT only should not be used as an indication for pregnancy termination [32]. Similar results were obtained by Yung et al. [33], showing a 50% PPV for positive SCA cases observed by NIPT in 170 out of 238 high-risk cases (47,890 total cases). The individual PPV was 70.58% for 47,XXX, 81.13% for 47,XYY/47,XXY, and 30% for 45,X0 [33].

In a study conducted by Reiss et al. [34], NIPT had a false positive rate of 91% (10/11 cases) and missed 4 true positive cases, all with cystic hygroma [34]. Suboptimal cell culture and fetoplacental and maternal mosaicism have been consistently reported as error sources in SCA detection [29,35,36].

Sun et al. [37] examined 3387 cases of pregnant women through cordocentesis, all of whom were considered at high risk of chromosomal abnormalities. A total of 182 abnormal karyotypes were identified, of which 37 cases were SCAs. A total of 16 of the 37 cases were sex chromosome mosaicisms. Equally, 4 out of 16 pregnant patients chose to continue the pregnancy, delivering 3 phenotypically normal children and 1 with ambiguous genitalia (46,X,i(Y)(q10) [20]/45,X [6]). Considering this outcome, the authors indicated that sex chromosome mosaicism should not be taken as a clear indication of pregnancy termination [37].

To differentiate between true SCA mosaicism and pseudomosaicism, novel techniques are being developed. Fan et al. [38] used segmental duplication quantitative fluorescent PCR (SD-QF-PCR) to show true mosaicism and mosaic proportions in 20 control samples and 14 amniotic fluid mosaic samples previously validated by first- and second-line karyotype analysis. Among the 14 mosaic samples, the numbers of samples showing true mosaicism and pseudomosaicism detected by this method were 6 and 8, respectively. Compared to karyotyping and FISH alone, this method can also be used on tissues from the chorionic villus or fibroblasts from aborted fetuses or stillborn babies, which would otherwise take a long time to cultivate. Mosaicism was completely detectable for proportions above 10%, but a larger sample size was considered necessary to validate the detection limit [38].

Zhang et al. [39] used BACs-on-Beads™ (BoBs) on 31 samples of amniotic fluid and umbilical cord blood from high-risk pregnancies that were already confirmed through karyotyping, single-nucleotide polymorphism microarray (SNP array), and copy number variation sequencing (CNV-seq) as cases of SCA mosaicism. Prenatal BoBs had a 74.2% sensitivity for detecting SCA mosaicism, with a detection limit of 6% [39].

Table 2 summarizes the positive predictive value of NIPT in detecting SCA.

## 4. Structural Chromosomal Mosaicisms

Structural chromosomal mosaicisms, which represent only a fraction of all chromosomal mosaicisms, can be identified by various methods, including karyotyping, chromosome microarray, SNP array, BACs-on-Beads (BoBs) assay, and multicolor FISH [40].

The use of the QF-PCR technique was also attempted, but it can only detect mosaicism when the abnormal cell line represents at least 10% of the whole sample and cannot detect inversions, translocations, or deleted/duplicated regions that are not within the STR marker range. A case study showed that the QF-PCR technique successfully identified the loss of the Yq11.2 region, subsequently confirmed by two additional techniques. Chromosome microarray analysis detected a 10.1 Mb deletion and a 16 Mb mosaic deletion (representing a mosaicism proportion of 20%) [16].

SNP array can provide important characteristics of complex mosaicisms, such as their content, origin, and mechanism. These details are significant for precise prenatal prognostic assessment and genetic counseling. A retrospective analysis of SNP array testing on 4512 prenatal diagnosis samples identified 15 cases of mosaic segmental duplication/deletion, involving both autosomal chromosomes and sex chromosomes. All of the cases resulted in intrauterine fetal death [41].

According to a study that included a total of 1409 pregnant women at high risk of chromosomal aberrations, the BoBs technique has a reduced detection rate for mosaicisms compared with karyotype analysis. More specifically, from a total of 6 cases of chromosomal mosaicism, 4 of them were missed by BoBs assay (including a case of a balanced translocation mosaicism) [42].

Among cytogenetic techniques, multicolor FISH (M-FISH) can detect mosaic complex chromosomal rearrangements when molecular techniques (MLPA, array-CGH) fail to give a conclusive result. This was the case in a clinical report that described the application of the M-FISH technique to finding the additional segment on chromosome 4 in a case of very low-level mosaicism for the cell line with the der(4)t(4;17) chromosome (5%). Only M-FISH led to the identification of the translocated DNA fragment, corresponding to additional chromosome 17q material on chromosome 4q [43].

Most cases of structural chromosomal mosaicisms detected prenatally have been described as deletions or duplications, as well as translocations at a lower frequency. A recent study using multiple techniques for the prenatal detection of chromosomal aberrations described a structural anomaly, which was found not to influence fetal development, most likely because the mosaicism was distinguished as a distal 5 p deletion in a single colony of amniocytes [44].

22q11.2 deletion syndrome (22q11.2DS) refers to a syndrome that results from the deletion of the 22q11.2 region, affecting approximately 1 in every 4000 to 6000 newborns and 1 in every 1000 unselected fetuses [43]. A rare case of 22q11 deletion syndrome (DS) was reported in China, where a healthy female with a history of two pregnancies with conotruncal defects was recruited in a clinical study. The mother had nearly none of the clinical characteristics that are related to 22q11.2 DS, except for a few insignificant abnormal results in her research facility examinations, such as hypocalcemia (2.21 mmol/L), hypophosphatemia (0.99 mmol/L), and a moderately low percentage of CD4+ T helper cells (percentage: 23.86%, absolute counts: 404.36 μL). It was supposed that the cell load with 22q11.2 deletion within the gonads may be higher than that in the blood. Performing interphase FISH, the results for the mother showed that 164 (82%) of her analyzed cells had normal signals and 36 (18%) of the cells presented a deletion of one copy of the 22q11.2 region. To confirm the 22q11.2DS mosaicism of the mother, metaphase FISH was performed, and hemizygous deletion signals were shown in more than 10% of the cells. The same structural chromosomal defect was also identified in the fetus, along with the presence of tetralogy of Fallot (ToF) and right renal agenesis, which was suggestive of multiple malformations. The pregnancy was interrupted, and labor was induced at approximately 28 weeks of gestation at the couple’s will [45].

A false positive result has proven to be a significant finding as part of the Dutch TRIDENT study. Using genome-wide non-invasive prenatal screening (NIPS), the research team detected a 20-megabase specific deletion starting at 10q25 in eight pregnancies. Since all the deletions started close to the *FRA10B* fragile site in 10q25, they investigated whether the pregnant women were indeed carriers of *FRA10B*. To confirm the presence of the deletion in the fetus, routine array analysis was performed on DNA isolated from amniotic fluid in four cases, but the deletion could not be confirmed in any of them. Therefore, they concluded that the result could have been caused by a maternal low-level mosaic deletion associated with *FRA10B* expansions, with no consequences for fetuses [46].

*Taiwanese Journal of Obstetrics & Gynecology* published a case of a chromosomal deletion involving the *NBEAP1* and *POTEB* genes located on chromosome 15 (15q11.1-q11.2), associated with diffuse lymphangiomatosis. Because of abnormal fetal ultrasound findings, a 33-year-old woman underwent amniocentesis at 22 weeks of gestation. Karyotyping revealed 46,XX,del(15)(q11.1q11.2)/46,XX mosaicism. The parents decided to have an abortion; therefore, it was not possible to obtain additional data on the evolution of the pregnancy [47].

Multiple Xq duplication syndromes have been reported, such as Opitz–Kaveggia syndrome, FG syndrome 5, Xq25 duplication syndrome, Xq26.3 duplication syndrome, Xq27.3-q28 duplication syndrome, and Xq28 duplication syndrome. A varying degree of intellectual disability or organic dysfunction characterizes each of these syndromes, but it was noted that a mosaic Xq duplication with the following chromosomal formula—46,X,der(X)dup(X)(q22.1q22.2)dup(X)(q25q22.3)/46,XX—may lead to pregnancies with a favorable outcome. Such was the case for a 40-year-old woman who underwent amniocentesis at 16 weeks of gestation due to her advanced maternal age. As a result of the culture artifacts, the in vitro culture process for the amniocytes caused an overestimated mosaic level, but the parents decided to continue the pregnancy, and a healthy female baby was delivered at 39 weeks of gestation [48].

Another case of structural mosaicism with a favorable outcome was reported as an association between a familial 8p23.2 microduplication encompassing the *CSMD1* gene and a balanced reciprocal translocation. A 38-year-old pregnant woman, with no phenotypical changes, underwent amniocentesis at 19 weeks of gestation due to her advanced maternal age. Amniocentesis revealed a karyotype of 46,XY,t(7;8)(q31.2;p23.1)/46,XY. It has been suggested that array comparative genomic hybridization (aCGH) is advisable in all carriers of balanced complex chromosomal rearrangements. aCGH analysis on the DNA extracted from both cultured amniocytes and parental blood cells revealed a 2.178 Mb 8p23.2 microduplication encompassing *CSMD1* in the fetus and the mother. At 38 weeks of gestation, a healthy male baby was delivered, with no developmental delays by the age of 6 months. All the analyzed cells in the cord blood, umbilical cord, and placenta had the karyotype of 46.XY [22].

A false positive result upon non-invasive prenatal testing due to a maternal structural chromosomal aberration has also been reported for duplications. A 37-year-old woman underwent amniocentesis at 19 weeks of pregnancy to ensure that fetal development was not affected by her advanced maternal age. Amniocentesis showed a karyotype of 46,XY. Simultaneous array comparative genomic hybridization (aCGH) revealed a 1.3 Mb duplication of 17p12. The mother did not have any phenotypical findings, but she also turned out to be a carrier of the same 17p12 microduplication. Prenatal ultrasounds found no anomalies, and the parents decided not to terminate the pregnancy [49].

Structural chromosomal mosaicisms involving translocations have been less frequently described in clinical studies. Prenatal diagnosis and postnatal follow-up in a case of mosaicism for a Robertsonian jumping translocation showed a favorable fetal outcome. Jumping translocations refer to a rare type of mosaicism in which the same chromosomal segment is translocated to different chromosomes in different cell lines. In this case, the newborn was a phenotypically normal male. The result of the first amniocentesis performed during the pregnancy was 45,XY,der(15;22)(q10;q10)/46,XY,i(15)(q10)/46,XY. The maternal karyotype was 45,XX,der(15;22)(q10;q10), and the paternal karyotype was 46,XY. The mother was referred for genetic counseling, and repeated amniocentesis performed at 23 weeks of gestation revealed 45,XY,der(15;22)(q10;q10)mat/45,XY,-22. Fluorescence in situ hybridization (FISH) analysis using a chromosome 15q-specific probe and a chromosome 22q-specific probe detected three 15q signals in 4/104 cells (3.8%), encompassing a trisomy. She was advised to continue the pregnancy, and it proved to be a case of transient mosaicism for a Robertsonian jumping translocation associated with a familial Robertsonian translocation, inherited from the mother by the child [50].

More information about the most common structural anomalies detected in mosaic forms can be found in Table 3.

## 5. Confined Placental Mosaicism

Confined placental mosaicism (CPM) is defined by the presence of two or more distinct cell lines with different chromosomal compositions within the placenta while the fetus maintains a uniform chromosomal formula. This can lead to complications in interpreting prenatal screening results, as the placental cells might not accurately represent the fetal genetic status. [54,55,56].

CPM typically arises early in embryonic development. Its causes are not fully understood, but there are several mechanisms by which it might occur, as summarized in Table 4:

Confined placental mosaicism (CPM) can be categorized into three main subtypes based on the location and type of chromosomal abnormalities within the placenta. These subtypes are essential for understanding the implications and origins of the mosaicism and are summarized in Table 5:

The detection of CPM typically involves chorionic villus sampling (CVS), which includes short-term culture of the villi and long-term culture of the villi and non-invasive prenatal testing (NIPT)—a positive NIPT finding is followed by CVS or amniocentesis. Studies show that NIPT is more sensitive to CPM compared to CVS. [56,58,59,60].

Because CPM affects only the placental chromosomal formula and not the fetal chromosomal number, chromosomal analysis of placental cells might not match the true chromosomal status of the fetus.

There are three main outcomes of CVS testing:

1. True positive: Both the placenta and the fetus have the same chromosomal abnormality.
2. True negative: Both the placenta and the fetus have normal chromosomes.
3. False positive/negative: The placenta shows a chromosomal abnormality that is not present in the fetus, or vice versa, indicating CPM.

Understanding the subtypes of CPM is crucial for assessing the potential impact on pregnancy and fetal development. The location and extent of mosaicism within the placenta determine the severity and implications of the condition.

Clinical studies show conflicting findings when it comes to how CPM influences pregnancies. Most pregnancies associated with CPM are uneventful, but some studies show that CPM can lead to fetal growth restrictions (FGRs). The exact type of CPM influences the placental function—types 2 and 3 are considered to have significant implications for FGRs, low weight at birth, or preterm delivery, being associated with adverse pregnancy outcomes such as low levels of first-trimester serum pregnancy-associated plasma protein A, preterm births, and newborns small for gestational age [61]. CPM is more common in spontaneous abortions than in viable pregnancies, and it is associated with an increased frequency of second- and third-trimester pregnancy loss or intrauterine fetal growth retardation [58,60,61]. The placental genome plays an important role in its development and optimal function. In cases of CPM, placental development and maternal–fetal exchanges may be affected; however, the precise mechanisms by which the placental genome impacts fetal development remain unclear [62].

The effects of CPM on the fetal phenotype and intrauterine development depend on the chromosomes involved and the distribution of abnormal cells among tissues. The most published placental abnormality is trisomy 16, which can have significant implications for pregnancy outcomes and fetal development. Placental trisomy 16 can be associated with intrauterine growth restriction and preeclampsia, miscarriage, and stillbirth due to its severe impact on placental function [63].

Other abnormalities associated with higher-risk pregnancies are trisomies 2, 3, 7, 13, 15, and 22. If a high-risk CPM is diagnosed through genetic testing, clinicians are advised to perform regular ultrasounds and fetal growth assessments [58,59,60,61,62,63,64,65].

Besides its effects on fetal development, CPM can also lead to false positive or false negative results in prenatal screenings: a positive NIPT test can result from a CPM and also from a fetal chromosomal abnormality. Genetic counseling is crucial to interpret the results, understand the implications, and guide the decision-making process [40,62,63,64,65]. In cases of suspected CPM, CVS or amniocentesis is the next step for validating the NIPT results, confirming the fetal genotype, and distinguishing between true fetal abnormalities and confined placental mosaicism. NIPT result validation through CVS or amniocentesis ensures that expectant parents receive accurate information, enabling them to make well-informed decisions about their pregnancy and prepare for any necessary medical interventions. It is important to acknowledge that amniocentesis and CVS, being invasive procedures, carry a small risk of infection, bleeding, or even miscarriage. However, in the context of CPM, the benefits of obtaining a definitive diagnosis often outweigh the risks [57].

## 6. Discussion

The development of advanced techniques, such as high-throughput sequencing, has significantly improved the standards of prenatal diagnosis [1]. This is particularly important in the context of genetic disorders, where prenatal and preimplantation genetic diagnosis can provide valuable information and the opportunity to make informed decisions regarding the outcome of the pregnancy. Up to 5% of pregnancies are affected by genetic disorders [66]. Among the most common chromosomal disorders found in prenatal diagnosis are chromosomal mosaicisms, with an incidence ranging from 0.5% to 0.6% [5].

Mosaicisms can affect human development and can lead to genetic abnormalities, miscarriages, stillbirths, or live births with various malformations but also healthy newborns with no phenotypical modifications. The clinical consequences of mosaicism depend on the chromosome involved, as well as the timing and location of the error [67]. Somatic mosaicism, a related concept, refers to the occurrence of two genetically distinct cell populations within an individual, derived from a postzygotic mutation. It has been found to be involved in various disorders, including cancer and neurodegenerative diseases [68]. The dynamic nature of chromosomal mosaicism throughout ontogeny and its association with human diseases highlight the need for further research in this area [69].

Chromosomal mosaicism in fetuses can lead to a range of outcomes, from severe microcephaly and growth deficiency to normal fetal growth and no evidence of intrauterine growth retardation. The prognosis appears to be more optimistic in cases without structural anomalies observed by ultrasound [5,70], although there have been cases reported with autosomal trisomy mosaicism showing that, when autopsied, the fetuses had several abnormalities that were not visible in the previous ultrasounds: facial dysmorphism, hypertelorism, intestinal malrotation, and partial anomalous pulmonary venous return [26]. However, the presence of mosaicism in embryos transferred during assisted reproductive technology (ART) can result in healthy babies, suggesting that the prognosis may vary depending on the specific chromosomal abnormalities and the extent of mosaicism [70]. An important factor for the phenotype is the mosaic fraction [15]. Further research is needed to fully understand the prognosis of fetuses with chromosomal mosaicism.

Confined placental mosaicism is a unique condition that underscores the complexity of prenatal genetics. Understanding the causes and implications of CPM is essential for accurate prenatal diagnosis and management. As prenatal testing technologies continue to advance, our ability to detect and interpret CPM will improve, ultimately enhancing the care and support provided to expectant parents [62,63,64].

While NIPT provides a high probability of detecting certain chromosomal abnormalities, it does not confirm them definitively. Positive results should be followed up with certain diagnostic tests, which implies obtaining fetal cells. Two primary methods for obtaining fetal cells for genetic analysis are amniocentesis and chorionic villi sampling (CVS). These procedures provide the material needed for various molecular genetic tests [71].

Amniocentesis can be performed between 15 and 20 weeks of pregnancy and involves collecting amniotic fluid, which contains fetal cells. These cells are cultured and then karyotyped. CVS is a diagnostic test that involves obtaining a small sample of placental tissue, which can provide more direct and detailed genetic information. CVS can be performed between 10 and 13 weeks of pregnancy, allowing for early diagnosis [72].

The genetic material obtained through amniocentesis or CVS can be analyzed using a variety of cytogenetic and molecular genetic tests. Each test has its specific applications, advantages, and limitations, and the choice of test depends on the clinical context and the specific information required (Table 6) [57,71,72,73,74].

In prenatal diagnosis, chromosomal karyotyping analysis and single-nucleotide polymorphism-based microarray (SNP array) are valuable for detecting chromosomal mosaicism in amniotic fluid samples, with FISH used for further verification [8]. The MrMosaic method, which uses deviations in the allele fraction and read coverage from next-generation sequencing data, has been developed to detect structural mosaic abnormalities [42]. High-resolution full-genome analysis methods, such as single-cell array-based comparative genomic hybridization, have been developed to address these challenges and have revealed significant fractions of cells with unique chromosomal abnormalities in human somatic and embryonic stem cell cultures [75].

Karyotyping plays a crucial role in the prenatal diagnosis of chromosomal mosaicism. For the past 50 years, it has been considered the golden standard for detecting chromosomal abnormalities [4]. It has been found to be an accurate and convenient method for detecting sex chromosome mosaicisms, which can help in making informed decisions about pregnancy continuation [76]. However, it is important to note that karyotyping has its limitations, and when used in combination with other techniques, such as single nucleotide polymorphism-based microarray (SNP array) and FISH, it can provide more precise information for genetic counseling [8]. Despite its sensitivity, karyotyping may also have limitations due to artifacts and bias resulting from cell cultivation, particularly for sex chromosomal abnormalities, where combining it with uncultured FISH or DNA-based methods are necessary [4,5].

Research has shown that the use of FISH can be valuable in the diagnosis of chromosomal mosaicism, especially in identifying mosaicism caused by postzygotic mutations and in characterizing small supernumerary marker chromosomes, respectively [77]. Also, the utility of FSIH in detecting low-level mosaicism for chromosomal rearrangements has been highlighted [78]. These studies collectively underscore the significance of FISH in the diagnosis of chromosomal mosaicism, particularly in cases where other molecular techniques may be insufficient [77,78].

Array analysis, particularly array comparative genomic hybridization (aCGH), has been shown to be effective in detecting chromosomal mosaicism in prenatal diagnosis [5,8,79,80]. This method has been used in over 1600 cases, with a high detection rate of mosaicism in chorionic villus and amniotic fluid samples [79]. It has also been compared to other techniques such as karyotyping and SNP array, with aCGH showing promising results [8]. Furthermore, a custom-designed, exon-targeted whole-genome oligonucleotide array has been used to detect somatic mosaicism in a significant number of cases [81]. Despite the challenges in detecting mosaicism, array analysis is beneficial in prenatal diagnosis, particularly when combined with other techniques [5].

Another method for analyzing chromosomal mosaicism is quantitative fluorescence polymerase chain reaction (QF-PCR). The main advantage of this method is the low cost and speed at which results are available (24–48 h) [16]. It is particularly useful for detecting trisomies 21, 18, and 13, as well as sex chromosome aneuploidies [16,81,82]. However, it may not be able to detect chromosomal rearrangements and some mosaic samples (when the normal cell line is below 10% of the whole sample), which require cytogenetic analysis [16,82].

NIPT is typically performed from around 10 weeks into a pregnancy and has been shown to have a high screening capacity for chromosomal abnormalities, particularly trisomy 21 (Down’s syndrome) [10]. The process of NIPT involves the quantification of copy number alterations from the sequencing of cell-free DNA, with various tools and software packages available for data analysis [83,84]. Despite its potential, the implementation of NIPT presents challenges, including the need for regulation and oversight, particularly in areas where sex-based abortions are prevalent [13].

NIPT has revolutionized prenatal diagnosis by enabling the detection of chromosomal mosaicism, a phenomenon that can lead to false positive and false negative results with traditional testing methods. The use of advanced bioinformatics algorithms in NIPT has allowed for the identification of fetoplacental mosaicism, which can influence risk estimation and improve genetic counseling [85]. Additionally, the development of a novel analysis pipeline for NIPT has improved the detection of all autosomal fetal aneuploidies, including mosaic trisomies, thereby enhancing prenatal management [86]. However, it is important to note that maternal mosaicism of sex chromosomes can cause discordant sex chromosomal aneuploidies in NIPT, highlighting the need for confirmatory testing [35].

**Table 6:** Genetic tests used in prenatal diagnosis [5,16,57,71,72,73,74,87,88,89,90,91,92].

Technique	Description	Indications	Genetic Defect Detected	Limitations
Karyotyping	The process of pairing and ordering all the chromosomes of an organism, providing a genome-wide snapshot of an individual’s chromosomes.	- advanced maternal age - abnormal maternal serum screening - known balanced translocations in the family - fetal abnormalities in ultrasound	- chromosome aneuploidies - balanced chromosomal rearrangements - deletions and duplications (>5–10 Mb) - chromosomal mosaicism	- artifacts, particularly for sex chromosomal abnormalities - long processing time - low resolution
Fluorescence In Situ Hybridization (FISH)	FISH uses fluorescent probes that bind to specific parts of the chromosome. This allows for the detection of specific genetic abnormalities.	- uncultured samples can be used - specific microdeletion suspected, too small to be identified on karyotyping	- chromosome aneuploidies - submicroscopic deletions and duplications - complex chromosomal rearrangements - low-level mosaicism for chromosomal rearrangements	- has a targeted approach - structural chromosomal aberrations can be missed - faster but less comprehensive than karyotyping
Quantitative Fluorescent Polymerase Chain Reaction (QF-PCR)	Rapid method for detecting aneuploidies by amplifying DNA regions containing specific short tandem repeats (STRs).	- quicker results than using conventional karyotyping	- aneuploidies - microdeletions/duplications	- detects chromosomal mosaicism when the abnormal cell line represents at least 10% of the whole sample - inversions, translocations, or deleted/duplicated regions must be within the STR marker range
Polymerase Chain Reaction (PCR)	Technique used to amplify small segments of DNA, allowing for detailed analysis.	- specific genetic conditions by targeting known mutations	- single gene mutations	- cannot be used to identify unknown targets - prone to errors during multiplication
Chromosomal Microarray Analysis (CMA)	CMA, also known as array comparative genomic hybridization (aCGH), detects copy number variations (CNVs) across the genome.	- low-risk pregnancies - fetuses with structural abnormalities - stillbirths	- deletions/duplications	- detects variants of unknown significance - does not detect balanced chromosomal rearrangements - low-level mosaicism can be missed
Multiplex Ligation Probe Amplification (MLPA)	MLPA is a technique used to detect CNVs and methylation abnormalities in specific genomic regions.	- uniparental disomies - imprinting errors - small deletions and duplications	- CNVs and methylation abnormalities in specific genomic regions	- cannot be used to identify unknown targets - prone to errors during multiplication
Next-Generation Sequencing (NGS)	NGS involves sequencing millions of small fragments of DNA in parallel, providing a comprehensive analysis of the genome.	- single-nucleotide mutations when not looking for a specific target region	- monogenic disorders - CNVs, small insertions/deletions	- can often detect variants of uncertain significance (VUSs) - expensive

As each situation will be unique, every diagnosed case of chromosomal mosaicism needs to be assessed carefully by clinicians. Genetic counseling plays a crucial role for these families, as it can provide the necessary information for them to decide how they proceed in the pregnancy and for future family planning [9]. Prenatal and postnatal phenotypical findings can vary significantly, even within the same genotype, and sometimes the phenotypes can present significant overlaps, even for completely different genotypes [93]. Although usually the exact prognosis for the patient is impossible to predict, proper diagnosis and checkups can provide the insights needed to make an informed decision about continuing or interrupting a pregnancy.

Low-level mosaicism is even harder to diagnose, especially when facing a rare chromosomal anomaly [9]. Special focus needs to be placed on the psychological impact on the families when genetic anomalies are discovered, and multidisciplinary teams are needed for the proper management of these cases, where the parents need to make informed decisions keeping in mind the potential quality of life of their future child [94].

## 7. Conclusions

Chromosomal anomalies, and especially mosaic variants, remain a great challenge for prenatal diagnosis, both for clinical geneticists and families. Proper genetic counseling is often difficult and often requires multiple discussions with family members. Wider access to innovative genetic testing will provide better opportunities for higher accuracy regarding prenatal testing results.

## Figures and Tables

**Table 1:** Genetic techniques and clinical impact in autosomal mosaicism cases [4,14,17].

Reference	No. of Cases	Tissue Sample	Analysis	Abnormal Type	Phenotype Severity
Ma et al. [4].	22 cases with autosomal aneuploidy mosaicism: 11 cases with T21 2 cases with T18 2 cases with T15 Other (T13, T9,T8, T22, T20, T2)	amniotic fluid or fetal cord blood	Karyotyping	Mosaicism (21 cases)	No information available
			CMA	Low-level mosaicism down to 20% (13 cases)	
			CNV-seq	Mosaicism level down to 5% (19 cases)	
Peng et al. [17]	14 cases with high risk of T16: 12 cases of T16 2 cases of T16 mosaicism	maternal blood	NIPT		
		amniotic fluid or cord blood	Karyotyping and CMA (following NIPT)	T16 mosaicism (five true positive cases)	Three cases had ultrasound abnormalities (a) Four fetuses died (induced labor, intrauterine death, or death after birth)
				Nine false positive cases	Eight babies had birth weights less than 2.5 kg (b) Two premature babies
Wang et al. [14]	Eight cases with fetal CR		NGS and ddPCR		Five fetuses presented single tumors Three fetuses presented multiple tumors One fetus had tricuspid regurgitation
	Six NMI cases with no previous variants of TSC1/TSC2	umbilical cord and CR tissue		Low-level mosaic variants (four cases)(c) Somatic mosaic variants in the CR tissue (two cases)(d)	
	Two cases suspected of familial gonadal mosaicism	umbilical cord, CR tissue, seminal fluid, and parental blood		TSC1/TSC2 gene variants (e)	

Abbreviations: T21, trisomy 21; T18, trisomy 18; T15, trisomy 15; T13, trisomy 31; T22, trisomy 22; T20, trisomy 20; T8, trisomy 8; T9, trisomy 9; fT2, trisomy 2; CMA, chromosomal microarray analysis; CNV-seq, copy number variation sequencing; NIPT, non-invasive prenatal testing; CR, cardiac rhabdomyoma; NMI, no mutation identified; NGS, next-generation sequencing; ddPCR, droplet digital polymerase chain reaction. (a) Ultrasound examination of case 1 revealed intrauterine growth restriction, a persistent right umbilical vein, and abnormal umbilical blood flow. The newborn had a low birth weight (1.9 kg), and no other abnormalities were present. In case 2, the ultrasound examination showed inconsistency with the gestational age, small limbs, and a cardiac defect. Echocardiography revealed total abnormal pulmonary venous drainage, a ventricular septal defect, and a left aortic arch with the right descending aorta. The newborn died due to congenital heart disease 13 days after birth. In case 3, the ultrasound scan showed a butterfly vertebral anomaly in T3, and the pregnancy was terminated. (b) In newborn screening, one case presented with cerebral edema and anemia. The mother had preeclampsia. (c) The allelic frequencies were higher within the cardiac rhabdomyoma tissue than in the umbilical cord tissue. (d) The allelic frequencies were absent in the umbilical cord tissue (0%). (e) Both fathers had low-level mosaicism and presented gonadal mosaic variants. The allele frequencies of these variants in the seminal fluid were higher than 30%.

**Table 2:** Total number, singular, and total PPV% obtained from cordocentesis of amniotic fluid for the most frequent SCAs [31,32,33,34,37].

Study	Cases Studied (nr.)	SCAs	45,X0	47,XXX	47,XXY	47,XYY	Mosaic SCAs	Other SCAs	Total PPV %
Dai et al. [31]	17.428	NIPT	41	11	17	10	12	-	38.46
		PPV (%)	28.59	36.36	37.50	57.14	83.33	-	
Xu et al. [32]	32.931	NIPT	62	29	28	20	0	One lower X case	55.34
		PPV (%)	26.09	85	85	68.75	-	0	
Yang et al. [33]	47.800	NIPT	137	27	47		-	-	50.00
		PPV (%)	30.00	70.58	81.13		-	-	
Reiss et al. [34]	2851	NIPT	11	5	2	0	0	0	44 * Large number of missed cases
		PPV (%)	9	100	100	-	-	-	
Sun et al. [37]	3.387	Cordocentesis	2	5	7	6	19	48,XXXX	

PPV—positive predictive value. * While for other SCAs the prediction was more accurate, the number of false positives for monosomy X was high (91%).

**Table 3:** Most common structural anomalies associated with mosaicism in prenatal diagnosis [45,46,47,51,52,53].

Structural Anomaly	22q11.2 Microdeletion	10qter Deletion	15q11.2 Deletion
Frequency	1 in 4000–6000 newborns, 1 in 1000 fetuses	1 in 1263	57–127 in 10,000
Type of tissue	amniotic fluid peripheral blood	amniotic fluid placental biopsies umbilical cord and blood maternal blood lymphocytes	amniotic fluid placental tissue umbilical cord maternal peripheral blood paternal peripheral blood
Type of test	amniocentesis fluorescence in situ hybridization (FISH) chromosomal microarray analysis	amniocentesis fluorescence in situ hybridization (FISH) array comparative genomic hybridization	amniocentesis array comparative genomic hybridization
Clinical implications	cardiovascular anomalies, immunodeficiency, endocrine abnormalities, renal abnormalities, developmental delays, and behavioral and mental disorders	facial dysmorphism, pre- and postnatal growth retardation, cardiac and genital anomalies, and developmental delay	neuropsychiatric or neurodevelopmental disorders dysmorphic features may be associated with diffuse lymphangiomatosis when involving the NBEAP1 and POTEB genes

**Table 4:** Possible causes of confined placental mosaicism [57,58].

Postzygotic Mutation	A mutation occurring in one of the early cell divisions can lead to two distinct cell lines: one with a normal set of chromosomes and one with an abnormal set. If the abnormal cells are confined to the placenta, CPM occurs.
Trisomy Rescue	Initially, the embryo might have three copies of a particular chromosome (trisomy). During subsequent cell divisions, one of these extra chromosomes might be lost in some cells. If this loss occurs predominantly in the fetal cell line, leaving the trisomic cells in the placenta, it results in CPM.
Placental Origin of Mutation	Sometimes, the mutation causing the chromosomal discrepancy occurs specifically in the trophoblast cells, which form the placenta, rather than in the cells destined to become the fetus. This leads to the placenta having a different chromosomal makeup than the fetus.

**Table 5:** CPM subtypes [55,56,57].

Subtype	Affected Placental Layer	Pregnancy Outcome
Subtype 1	This subtype involves mosaicism confined to the cytotrophoblast layer of the placenta. In this type, abnormal cells are present only in the cytotrophoblast, which is the outer layer of the placenta that makes direct contact with maternal blood.	Subtype 1 CPM often has less impact on fetal development because the abnormal cells do not infiltrate into the chorionic villi where nutrient exchange primarily occurs.
Subtype 2	This subtype includes mosaicism confined to the mesenchymal core of the chorionic villi. Abnormal cells are found within the mesenchymal (connective tissue) core of the placental villi but not in the cytotrophoblast.	Subtype 2 CPM might have more significant implications for fetal development because it can affect the structural integrity and function of the placental villi, potentially leading to issues like intrauterine growth restriction (IUGR).
Subtype 3	This subtype involves mosaicism in both the cytotrophoblast and the mesenchymal core of the chorionic villi. Abnormal cells are present in both major compartments of the placenta, indicating a widespread distribution of mosaicism.	Subtype 3 CPM is generally considered the most severe form, with a higher likelihood of impacting fetal development and pregnancy outcomes due to the extensive involvement of the placenta.
